# Mitochondrial genomes of five *Hyphessobrycon* tetras and their phylogenetic implications

**DOI:** 10.1002/ece3.8019

**Published:** 2021-08-11

**Authors:** Wei Xu, Shupeng Lin, Hongyi Liu

**Affiliations:** ^1^ College of Biology and the Environment Nanjing Forestry University Nanjing China

**Keywords:** Characidae, *Hyphessobrycon*, Mitochondrial genome, molecular phylogeny, tandem repeat

## Abstract

To date, the taxonomic status and phylogenetic affinities within *Hyphessobrycon*, even among other genera in Characidae, remain unclear. Here, we determined five new mitochondrial genomes (mitogenomes) of *Hyphessobrycon* species (*H. elachys*, *H. flammeus*, *H. pulchripinnis*, *H. roseus*, and *H. sweglesi*). The mitogenomes were all classical circular structures, with lengths ranging from 16,008 to 17,224 bp. The type of constitutive genes and direction of the coding strand that appeared in the mitogenomes were identical to those of other species in Characidae. The highest value of the Ka/Ks ratio within 13 protein‐coding genes (PCGs) was found in ND2 with 0.83, suggesting that they were subject to purifying selection in the *Hyphessobrycon* genus. Comparison of the control region sequences among seven *Hyphessobrycon* fish revealed that repeat units differ in length and copy number across different species, which led to sharp differences in mitogenome sizes. Phylogenetic trees based on the 13 PCGs did not support taxonomic relationships, as the *Hyphessobrycon* fish mixed with those from other genera. These data were combined to explore higher level relationships within Characidae and could aid in the understanding of the evolution of this group.

## Graphical abstract

To date, the taxonomic status and phylogenetic affinities within *Hyphessobrycon*, even among other genera in Characidae, remains unclear. Here, we determined five new mitochondrial genomes of *Hyphessobrycon* species.

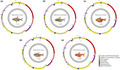

## 1 INTRODUCTION

Characidae is the most diverse family of tropical fish, with approximately 163 genera and more than 1,050 valid species, of which 231 have been described in the last 10 years. This family richness accounts for about 52% of all species in the order (Mirande, 2019; Paz et al., 2014; Veríssimo‐Silveira et al., 2010). Most members of the Characidae are small‐sized fish of <8 cm in standard length, reaching as much as 20 cm in some predatory genera. Fish of this family are characterized by a small adipose fin on the caudal peduncle, and most species have small, beautiful bodies and gentle temperaments (Mathubara & Toledo‐Piza, 2020). Characidae is one of the most popular ornamental fish groups in the world, with great economic value (Mirande, 2019; Sun et al., 2021). Many fish of the family are known in the aquarium market under the popular name of “tetras” (Camacho et al., 2020; Leggatt & Devlin, 2020; Liu, Sun, et al., 2020; Liu, Xu, et al., 2020; Paz et al., 2014). Until now, the classification of tetra fish is mainly based on morphological characteristics. Indeed, molecular phylogeny might differ from morphological classification within tetra fish (Liu, Sun, et al., 2020; Liu, Xu, et al., 2020; Mirande, 2019).

The high diversity of Neotropical freshwater fish has been challenging to classify (DoNascimiento et al., 2017; Mirande, 2019; Silva et al., 2020). Although the complex biogeographic patterns of some of these taxa (extending over vast continental areas) have been the focus of much research recently, Characidae, which have small body size and relatively uniform morphology, are still poorly understood. Furthermore, new genera and species in this family are being validated and described (Albornoz‐Garzon et al., 2019; DoNascimiento et al., 2017; Faria, et al., 2020; Faria, et al., 2020; Mathubara & Toledo‐Piza, 2020). *Hyphessobrycon*, one of the richest genera of vertebrates with 109 species, is the most diverse fish genus that dominates vertebrate neotropical freshwater. Native to the Neotropics, *Hyphessobrycon* is widely distributed from southern Mexico to Argentina (Rio de la Plata), with the greatest species diversity found in the Amazon River basin (Faria, Bastos, et al., 2020; Faria, Lima, et al., 2020; Paz et al., 2014). Classifying the genus and even the entire family of Characidae is currently challenging.

The mitochondrial genome (mitogenome) is a highly conserved, typically double‐stranded, circular molecule. In vertebrates, the mitogenome is approximately 15–18 kb in length and consists of 13 protein‐coding genes (PCGs), 2 ribosomal RNAs (rRNAs), and 22 transfer RNAs (tRNAs). The outer ring is a heavy chain, while the inner ring is a light chain, and most genes are transcribed by the heavy chain (Bernt, Braband, et al., 2013; Bernt, Donath, et al., 2013; Kurabayashi & Ueshima, 2000; Liu, Sun, et al., 2020; Liu, Xu, et al., 2020). Because of their small size, simple structure, low level of recombination, maternal inheritance, relatively high evolution rate, and conserved gene components, the mitogenome has been one of the most popular tools widely applied in taxonomy, population genetics, and evolutionary biology (Brown et al., 1979; Ciloglu et al., 2020; Sharma et al., 2020; Zhang, Gao, et al., 2020; Zhang, Sun, et al. 2020).

Considering the limited research using molecular data to infer taxonomic relationships, it is necessary to make comprehensive comparisons of morphological and genetic features of many species, to better understand the phylogenetic relationships with Characidae (Mirande, 2019). Our study on five new mitogenomes of *Hyphessobrycon* will help to improve the current classification of tetra fish by comparing the differences between mitogenomes of fish belonging to the same genus. Specifically, the mitochondrial characteristics of these five species, including gene order, genome size, nucleotide composition, codon usage, tRNA secondary structure, and noncoding control region (CR), were comparatively analyzed. This study provides new insights into the phylogeny and classification of tetra fish.

## 2 MATERIALS AND METHODS

### 2.1 Ethics statement

The collection and sampling of the specimens were reviewed and approved by Nanjing Forestry University. All specimens for this study were collected in accordance with Chinese laws. All the experiments were performed with animal welfare and care.

### 2.2 Sample collection and DNA extraction

Five *Hyphessobrycon* fish, including *Hyphessobrycon elachys*, *Hyphessobrycon flammeus*, *Hyphessobrycon pulchripinnis*, *Hyphessobrycon roseus*, and *Hyphessobrycon sweglesi*, were used for this study. These fish were bought from Nanjing Pet Market (Nanjing, China). The tail fin of each specimen was cut off after morphological identification described by the FishBase (available at https://www.worldfishcenter.org/fishbase). Genomic DNA (gDNA) was isolated from each fin using a FastPure Cell/Tissue DNA Isolation Mini Kit (Vazyme™, Nanjing, China) according to the manufacturer's protocol. The concentration and integrity of gDNA were tested using gel electrophoresis. High‐quality gDNAs were then stored at −20°C for future experiments.

### 2.3 PCR amplification and sequencing

According to the already published mitogenomes of Characidae species (*H. herbertaxelrodi*: MT769327.1, *H. megalopterus*: MT185596.1, *Hemigrammus bleheri*: LC074360.1, and *Gephyrocharax atracaudatus*: MH636341.1), ten pairs of universal primers were designed for PCR amplification (Table 1). PCR was performed with Taq Master Mix (Vazyme, Nanjing, China) under the following conditions: 3 min initial denaturation at 95°C, followed by 35 cycles of 30 s at 95°C, 30 s at 55–60°C, and 1–3 min at 72°C, and a final elongation for 5 min at 72°C. After gel electrophoresis, PCR products were sent to the TSINGKE Biological Technology (Nanjing, China) for Sanger sequencing.

**TABLE 1:** The primers used for mitochondrial amplification

Primer name	Region	Primer sequence (5′→3′)
Hyp‐1‐F Hyp‐1‐R	12S‐ND1	F: TGCTTAATATTACATATGGA R: CCGATTCAGGCTAGCAATCA
Hyp‐2‐F Hyp‐2‐R	ND1‐ND2	F: ACGAGAAGACCCTATGGAGC R: GACCAAGYTCTGCCCGGA
Hyp‐3‐F Hyp‐3‐R	ND2‐COXⅠ	F: TATCCCATATCTTCTGAATG R: GCAATTAGTGATATTAAGG
Hyp‐4‐F Hyp‐4‐R	COXⅠ‐COXⅡ	F: TTCTGCTTCTTTCTTCCGAT R: ACAGCCAATTTAACAGCCGG
Hyp‐5‐F Hyp‐5‐R	COXⅡ‐ ATP6	F: GAACATATGAATACACGGAC R: ACAAAGACGTATGCTTGAAT
Hyp‐6‐F Hyp‐6‐R	ATP6‐COXⅢ	F: GGGATACGAAACCAACCAAC R: GTATCAGGCGGCTGCCTCAA
Hyp‐7‐F Hyp‐7‐R	COXⅢ‐ND5	F: TACTTAACCTTGGTTAGAGC R: GAGGTGTTTAGGGCTTCAA
Hyp‐8‐F Hyp‐8‐R	ND5‐ND6	F: CAACCTCAACTAGCATTTAT R: CCTATTTTTCGGATATCTTG
Hyp‐9‐F Hyp‐9‐R	ND6‐Cytb	F: CCTGCTGGTGTAGCTTAACC R: GCCTTGTTGTTTTGATGTGTG
Hyp‐10‐F Hyp‐10‐R	Cytb‐16S	F: GCCTACGCCATCCTCCGATC R: TGGGTCAGGTTGTGTCCTGG

### 2.4 Sequence analysis

Sequences were spliced using DNAstar V.7.0. The BLAST CD‐search (available at https://www.ncbi.nlm.nih.gov/Structure/cdd/wrpsb.cgi) and MITOS Webserver (available at http://mitos.bioinf.uni‐leipzig.de/index.py) were used to detect conserved domains (Benson et al., 2018; Bernt, Braband, et al., 2013; Bernt, Donath, et al., 2013; Burland, 2000). The gene maps of these fish mitogenomes were generated with the OGDRAW Server (available at https://chlorobox.mpimp‐golm.mpg.de/OGDraw.html) (Greiner et al., 2019). The formulas “AT‐skew = (A − T)/(A + T)” and “GC‐skew = (G − C)/(G + C)” were used to measure nucleotide bias (Perna & Kocher, 1995). Codon usage and amino acid composition were analyzed in MEGA Ⅹ and image rendered by PhyloSuite v1.2.1 (Kumar et al., 2018; Zhang, Gao, et al., 2020; Zhang, Sun, et al., 2020). The rate of nonsynonymous substitutions (Ka), rate of synonymous substitutions (Ks), and ratio of Ka/Ks were determined using DnaSP V.6.0 for these five *Hyphessobrycon* species (Rozas et al., 2017). tRNA genes were identified using tRNAscan‐SE Search Server (available at http://lowelab.ucsc.edu/tRNAscan‐SE/) (Chan & Lowe, 2019). Some tRNAs not detected by tRNAscan‐SE were determined in the unannotated regions by sequence similarity to tRNAs of other fish.

### 2.5 Phylogenetic analyses

In addition to five newly sequenced mitogenomes, 33 species from 26 genera of Characiformes, *Cyprinus carpio* from Cypriniformes, and *Lateolabrax japonicus* from Perciformes were used for phylogenetic analyses. Their accession numbers and information are listed in Table S1. After sequence alignment and model prediction using MAFFT v7.313 and ModelFinder, phylogenetic analyses were conducted for each dataset using Bayesian inference (BI) and maximum likelihood (ML) methods available in the PhyloSuite v1.2.1 (Kalyaanamoorthy et al., 2017; Katoh & Standley, 2013; Zhang, Gao, et al., 2020; Zhang, Sun, et al., 2020). BI analyses were performed with MrBayes 3.2.6 (Huelsenbeck & Ronquist, 2001) and run for a million generations, with tree sampling every 100 generations and a burn‐in of 25% trees, while ML analyses were performed using the TIM2+F+R5 model in the IQ‐TREE (Nguyen et al., 2015). Clade support was assessed using a nonparametric bootstrap with 1,000 replicates, and phylogenetic trees were viewed and edited in iTOL (available at https://itol.embl.de/) (Letunic & Bork, 2021).

## 3 RESULTS AND DISCUSSION

### 3.1 Genome organization and base composition

**FIGURE 1:**
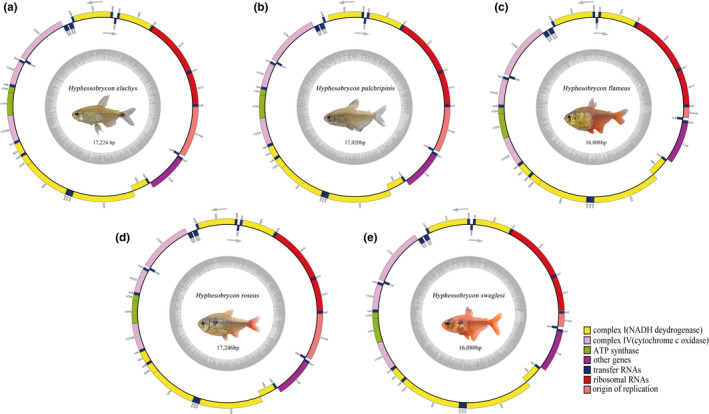
Mitochondrial genomes of *Hyphessobrycon elachys* (a), *Hyphessobrycon flammeus* (b), *Hyphessobrycon pulchripinnis* (c), *Hyphessobrycon roseus* (d), and *Hyphessobrycon sweglesi* (e)

The complete mitogenomes of the five fish were typically circular, double‐stranded molecules: 17,224 bp long for *H. elachys*, 16,008 bp for *H. flammeus*, 17,020 bp for *H. pulchripinnis*, 17,046 bp for *H. roseus*, and 16,080 bp for *H. sweglesi* (Figure 1). Among these fish, *H. flammeus* had the smallest mitochondrial genome with 16,008 bp, while *H. elachys* had the largest (17,224 bp) due to large‐scale duplication. Mitogenomes of the five fish encoded all 37 typical mitochondrial genes (13 PCGs, 22 tRNAs, and 2 rRNAs) and one noncoding CR. Twenty‐six genes were transcribed from the majority strand (J strand), and the remaining nine genes were from the minority strand (N strand) in these five mitogenomes. The gene orders of the five fish (Figure 1; Table S2) were found to be identical to those of two other species of this genus that have been previously sequenced (Liu, Sun, et al., 2020; Liu, Xu, et al., 2020; Yan et al., 2020).

**FIGURE 2:**
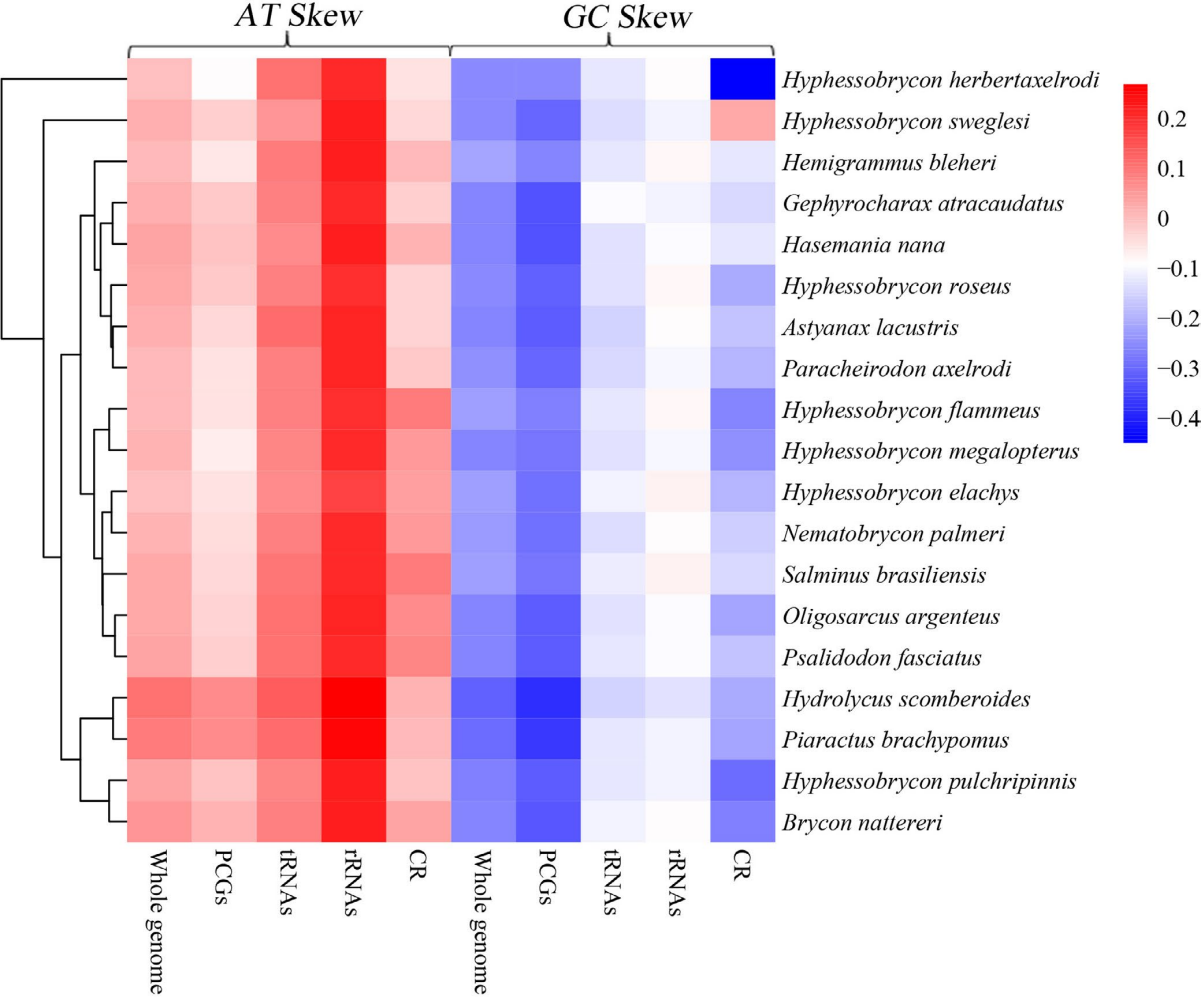
Nucleotide composition of various datasets of mitogenomes. Hierarchical clustering of Characidae species (*y*‐axis) based on the AT‐skew and GC‐skew

The skewness of the base composition in nucleotide sequences was used to measure the relative numbers of A to T (AT‐skew) and G to C (GC‐skew), and the nucleotide compositions of 19 complete or nearly complete mitogenomes in Characidae were investigated by calculating the percentages of AT‐skew and GC‐skew (Figure 2). The results of the nucleotide skew statistics showed that the AT‐skews in PCGs, tRNAs, and rRNAs of five whole mitogenomes were almost all positive, while the GC‐skews were all obviously negative. The low GC‐skew values among the analyzed mitogenomes (−0.269 – −0.221) indicated the occurrence of more Cs than Gs, which was also observed in other announced Characidae fish mitogenomes. The pattern of nucleotide skewness in *Hyphessobrycon* mitochondrial genomes is consistent with those of most other Characidae (Xu et al., 2015; Brandão‐Dias et al., 2016; Chen et al., 2016; Li et al., 2016; Zhang et al., 2016; Barreto et al., 2017; Isaza et al., 2016; Liu et al., 2019; Liu, Sun, et al., 2020; Liu, Xu, et al., 2020; Yan et al.,^2020^).

Eight gene overlaps were observed in the *H. elachys* mitogenome with sizes ranging from 2 to 15 bp, adding up to 32 bp; 10 gene overlaps in the *H. flammeus* mitogenome with sizes ranging from 2 to 14 bp, adding up to 35 bp; 10 gene overlaps in the *H. pulchripinnis* mitogenome with sizes ranging from 1 to 14 bp, adding up to 33 bp; 10 gene overlaps in the *H. roseus* mitogenome with sizes ranging from 1 to 11 bp, adding up to 27 bp, and 8 gene overlaps in the *H. roseus* mitogenome with sizes from 1 to 14 bp, adding up to 31 bp (Table S2). Additionally, the longest overlap region (15 bp) in the five mitogenomes was located between ATP8 and ATP6. All five species have two identical overlap regions including COXI‐tRNA‐Ser (11 bp) and ND4L‐ND4 (5 bp, except for *H. elachys* with 4 bp).

### 3.2 PCGs and codon usage

PCG lengths of the mitogenomes were 11,806 bp for *H. elachys*, 11,438 bp for *H. flammeus*, 11,355 bp for *H. pulchripinnis*, 11,450 bp for *H. roseus*, and 11,461 bp for *H. sweglesi*, accounting for 68.5%, 71.5%, 66.7%, 67.2%, and 71.3% of their entire make‐ups, respectively (Figure 1; Table S2). One PCG (ND6) was transcribed from the N strand, while the remaining 12 genes were from the J strand (Figure 1 and Table S2). The sizes of 13 PCGs ranged from 165 (ATP8) to 1,840 bp (ND5) in these five mitogenomes. All mitogenomes showed similar characteristics including the smallest size of ATP8 and the largest size of ND5 among PCGs.

Almost all PCGs in the five newly sequenced mitogenomes start with the standard ATG codon, except ATP6 in *H. sweglesi* that starts with the CTG codon (Table S2). Two other unusual initiation codons, TTG and GTG, have previously been reported in Characidae (such as in *Paracheirodon innes*: KT783482.1, *Astyanax paranae*: KX609386.1, and *Oligosarcus argenteus*: MF805814.1) (Silva et al., 2016). Furthermore, four termination codons were found in the PCGs of the five mitogenomes, namely TAA, TAG, AGG, and T (Table S2). In all mitogenomes, the occurrence frequency of the termination codon TAA was higher than those of the other three termination codons, while the termination codon AGG occurred the least.

**FIGURE 3:**
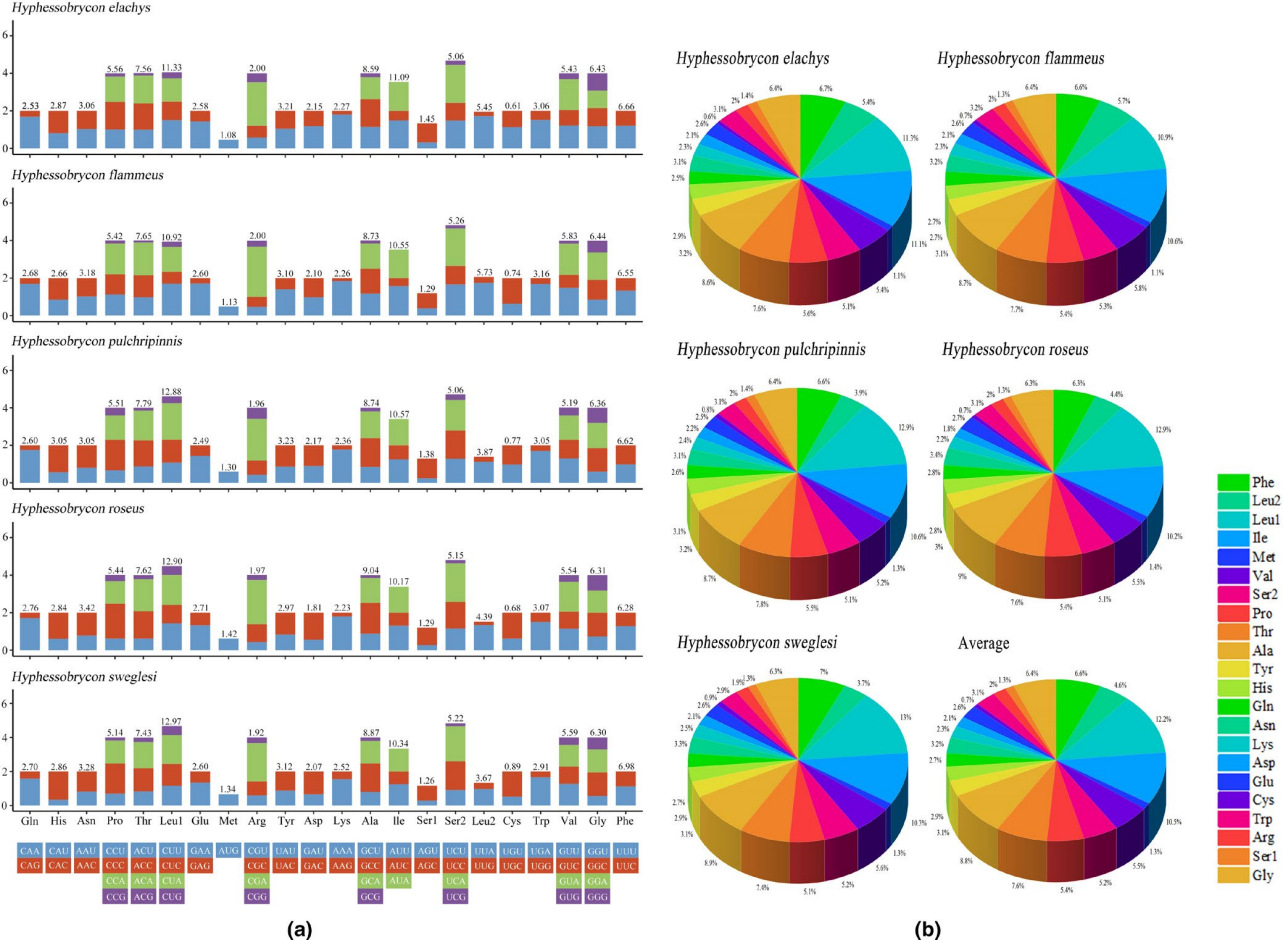
RSCU (a) and numbers of different amino acids (b) of the mitogenomes of five species of Characidae. The stop codon is not included

**FIGURE 4:**
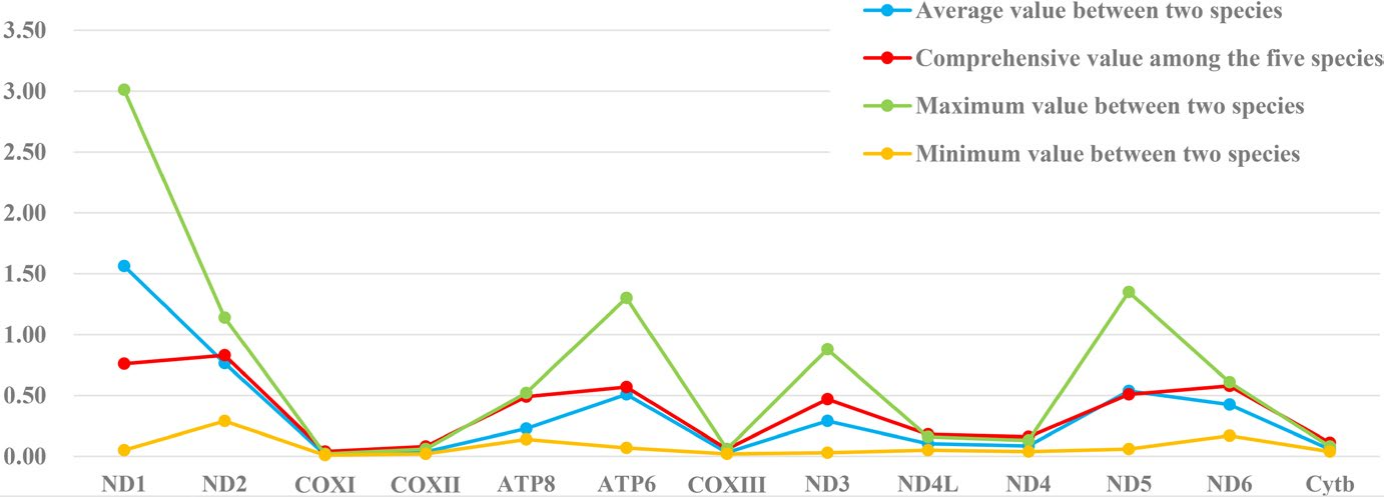
The maximum, minimum, and average Ka/Ks values between pairs of five species and the comprehensive values among the five species

Summaries of the relative synonymous codon usage (RSCU) and number of amino acids in 13 PCGs were calculated for the five mitogenomes, as shown in Figure 3. The amino acid compositions and RSCUs of these mitogenomes were found to be largely similar. Further, we calculated Ka/Ks ratios for each PCG of these mitogenomes, as shown in Figure 4, and Ka/Ks ratios of five species were compared in turn with each other. In evolutionary analysis, it is necessary to understand the rate at which Ks and Ka mutations occur, analyzing their ratios to detect selective pressures, if any, among PCGs. In this study, the PCGs of the assessed five species evolved under purifying selection as a whole, with COX I and COX II having the lowest evolutionary rate, and ND1 having the highest sequence variability. The Ka/Ks values of *H. elachys* and *H. flammeus* (0.05) were much lower than those of *H. elachys* or *H. flammeus* and the other three species (range: 2.10–3.01) in ND1.

### 3.3 rRNA and tRNA genes

Two rRNA genes (12S and 16S rRNAs) were transcribed from the J strand in the five mitogenomes. The large rRNA (16S rRNA) was found between tRNA‐Val and tRNA‐Leu, while the small rRNA (12S rRNA) was located between tRNA‐Phe and tRNA‐Val. Lengths ranged from 945 to 952 bp in 12S rRNA and from 1,669 to 1,679 bp in 16S rRNA in the mitogenomes.

**FIGURE 5:**
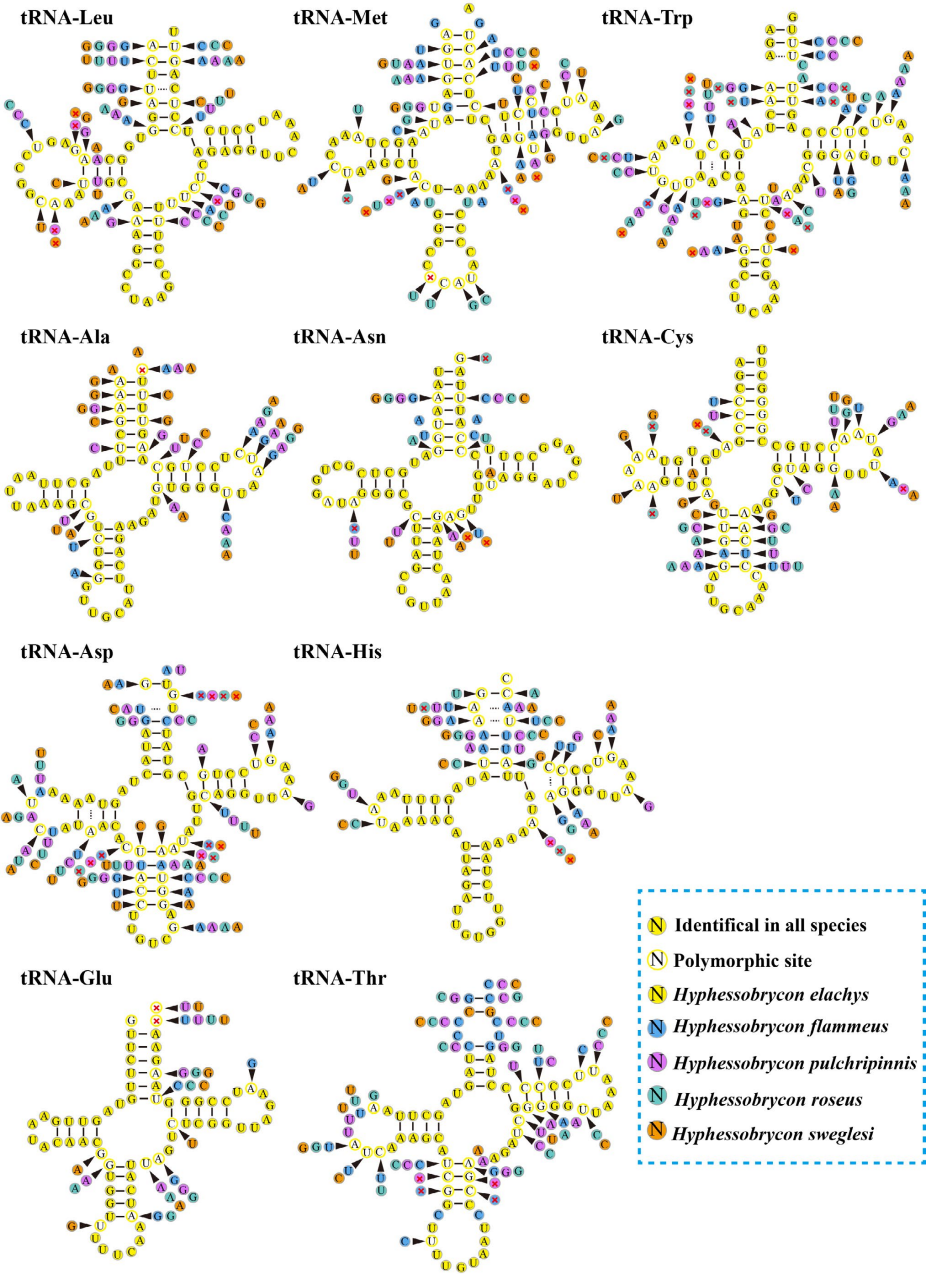
The secondary structures of ten groups tRNAs with significant differences among the five mitogenomes

Twenty‐two tRNAs of *H. elachys*, *H. flammeus*, *H. pulchripinnis*, *H. roseus*, and *H. sweglesi* mitogenomes were scattered discontinuously over the entire mitogenome (Table S2). The tRNA regions of these five mitogenomes were 1,556, 1,555, 1,561, 1,558, and 1,559 bp, accounting for 9.0%, 9.7%, 9.2%, 9.1%, and 9.7% of the whole mitogenomes, respectively. These five mitogenomes have 22 typical tRNA genes, with eight transcribed from the N strand and 14 from the J strand. The sizes of these tRNAs ranged from 68 to 74 bp. Except for tRNA‐Phe of *H. sweglesi*, all the tRNAs could be folded into secondary structures. The peculiar structures of tRNAs have also been reported in previous studies (Yuan et al., 2015). The ten most diverse tRNAs of all the five genomes are shown in Figure 5. Except for the classic AU and CG pairs, a number of mismatched base pairs were found in different stems. Fifteen AC mismatches, 10 UU mismatches, 8 CU mismatches, 3 AA mismatches, 3 CC mismatches, 1 AG mismatch, 1 GG mismatch, 2 extra single A, 2 extra single U, 1 extra single C, and 1 extra single G nucleotide were found in these ten groups of tRNAs.

### 3.4 CR

**FIGURE 6:**
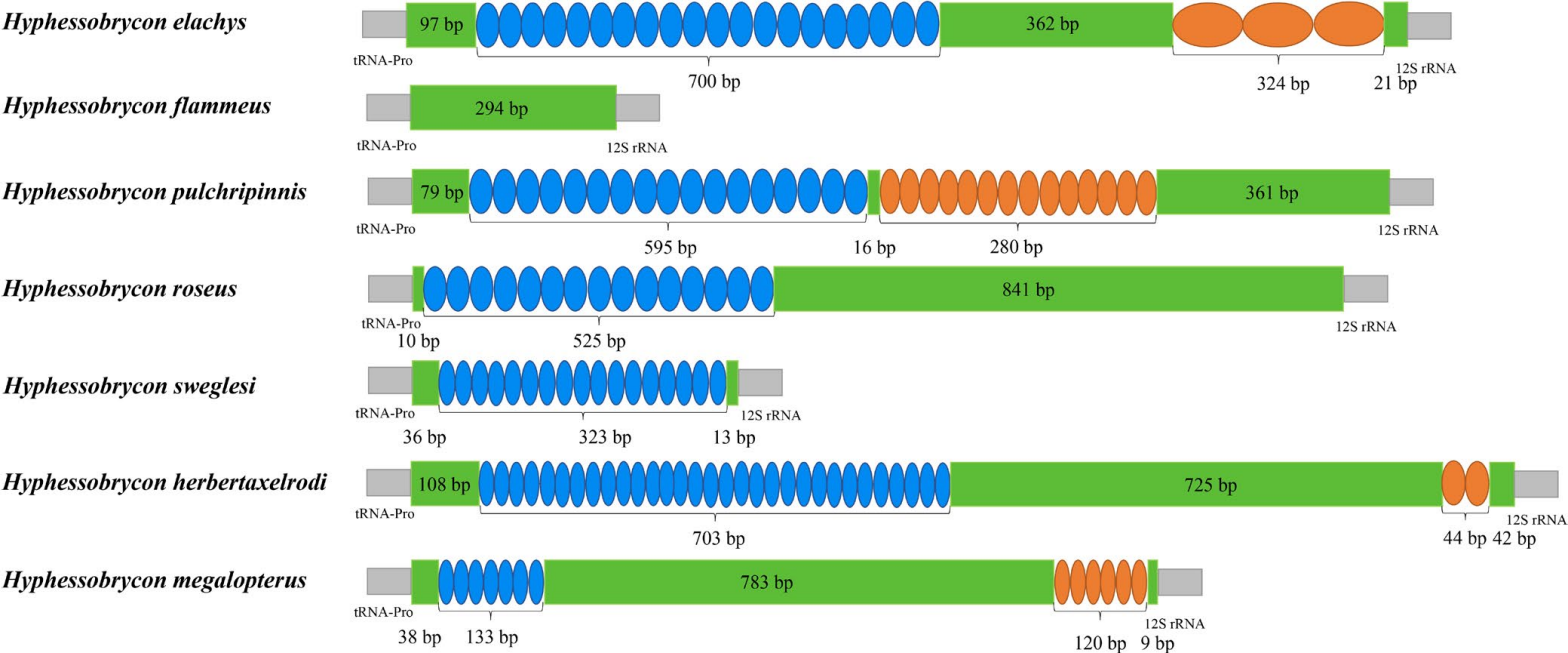
Organization of the control region in seven *Hyphessobrycon* mitochondrial genomes. The colored ovals indicate the tandem repeats; the remaining regions are shown with green boxes

CR is located between the genes tRNA‐Pro and tRNA‐Phe. This region is responsible for regulating transcription and replication. A + T contents in the CRs of the five mitogenomes were 67.7%, 76.2%, 73.2%, 66.4%, and 78.4%, respectively. According to previous reports, the CRs of fish vary significantly between different species and even within the same species (Buroker et al., 1990; Cui et al., 2010; Gong et al., 2015; Padhi, 2014). Among *Hyphessobrycon* fish, *H. flammeus* had the smallest CR length with 294 bp, while *H. herbertaxelrodi* had the largest with 1,622 bp (Figure 6). Further, *H. elachys* had the longest tandem repeats with a size of 1,024 bp, while the other species have relatively small sizes ranging from 353 to 875 bp. The repeat units differ in length and copy number across various *Hyphessobrycon* species. Hence, the evident differences in CRs were mainly caused by repeat units.

### 3.5 Phylogenetic relationships

**FIGURE 7:**
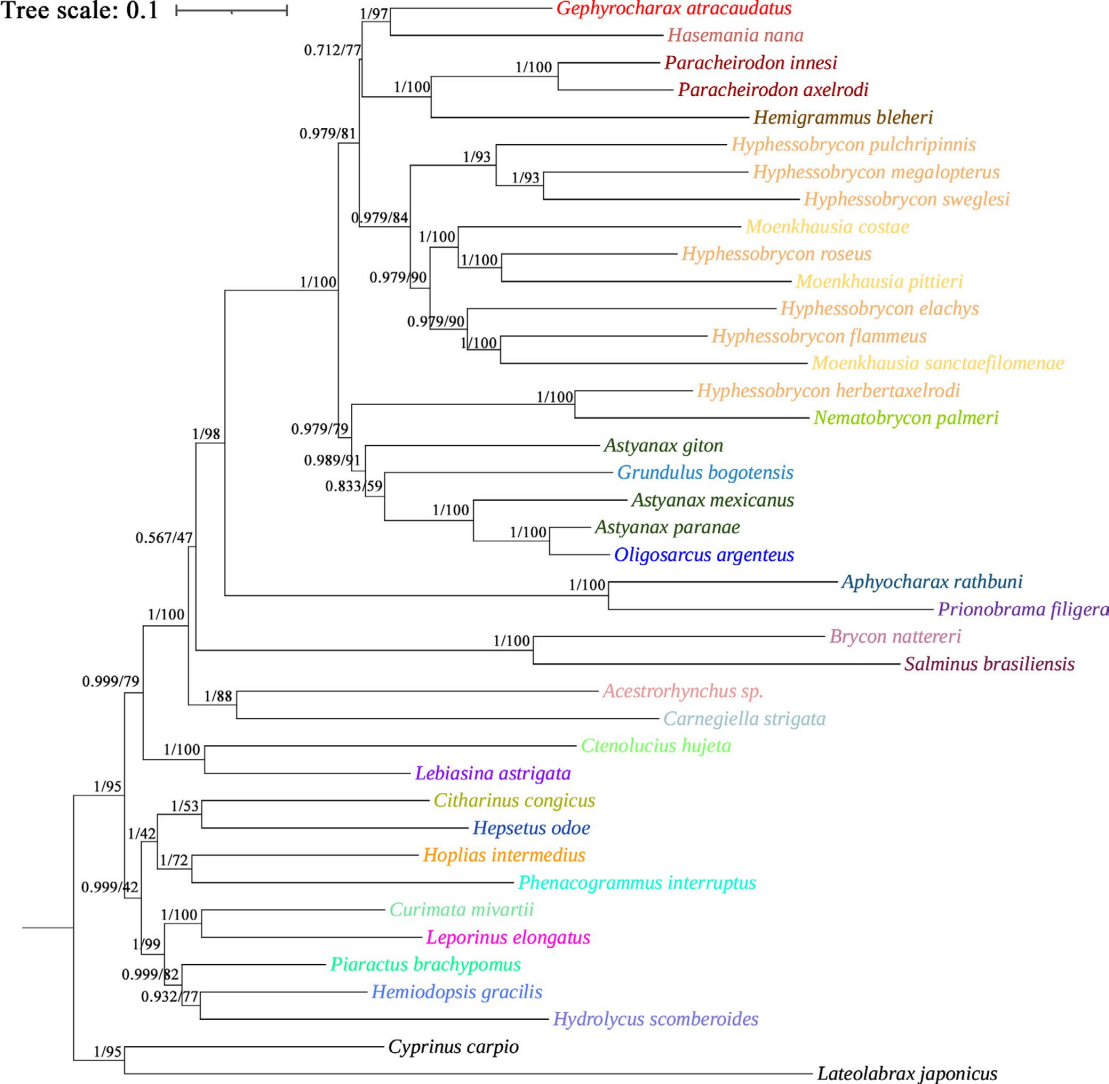
Phylogenetic tree produced by BI and ML based on the PCGs. Numbers at nodes are statistical support values for BI (posterior probabilities)/ML (bootstrap values)

Because of the limited mitogenome sequences of Characidae, we included only 33 species in addition to the five newly sequenced species from 26 genera of Characiformes in the phylogenetic analyses and selected two fish among Cypriniformes (*C. carpio*) and Perciformes (*L. japonicus*) as outgroups to root the phylogenetic tree to understand the evolutionary relationships of *Hyphessobrycon* with other genera within the Characidae family. Phylogenetic trees of BI and ML analyses were constructed based on 13 PCG nucleotide sequences from 40 species (Figure 7). The topological structures of the resulting trees were exactly similar to each other. Most nodes were rather highly supported (PP > 0.567 in BI analyses and also supported in the ML tree). According to the phylogenetic tree, these mitogenomes of Characidae were classified together. The existing taxonomic classification of Characidae on NCBI (https://www.ncbi.nlm.nih.gov/taxonomy) and ITIS (https://www.itis.gov/) is different. In this article, we mainly classified them according to the phylogenetic tree based on complete mitogenomes. Recent molecular hypotheses suggested that some traditional suprageneric taxa of Characiformes require revision. Serrasalmidae and Bryconidae, traditionally regarded as the subfamilies of Characidae, have been suggested to be recovered (Abe et al., 2014; Calcagnotto et al., 2005; Mirande, 2009). This point is also supported in the phylogenetic tree of this paper. *Piaractus brachypomus* did not cluster with species from the Characidae, but with species from other families. *Brycon nattereri* and *Salminus brasiliensis* clustered together but not with the Characidae species. *H. pulchripinnis* and *H. sweglesi* were classified together, while *H. elachys*, *H. flammeus*, and *H. roseus* were classified with other species of different genera and slightly away from the other fish of the same genus, which showed similarity to previous reports (Guimarães et al., 2019, 2020). Although the species in *Paracheirodon* were classified together, *Astyanax* and *Hyphessobrycon* species were not classified together, indicating that there may be some problems with the basis of classification. The result was indeed quite different from the existing classification system, although it only involved 27 genera in Characiformes. Additionally, we found that many species of the same genus have large differences in morphological characteristics, while species from different genera have similar morphological characteristics, which we hope to verify in future studies.

## CONFLICT OF INTEREST

All the authors declared no potential interest.

## AUTHOR CONTRIBUTIONS

**Wei Xu:** Formal analysis (equal); Funding acquisition (equal); Writing‐original draft (equal). **Shupeng Lin:** Formal analysis (equal); Funding acquisition (equal). **Hong‐Yi Liu:** Conceptualization (equal); Supervision (equal); Writing‐review & editing (equal).

## Supporting information

Table S1

Table S2

## Data Availability

DNA sequences: GenBank accession number MW315747 for *H. elachys*, MW315748 for *H. flammeus*, MW315750 for *H. pulchripinnis*, MW315749 for *H. roseus*, and MW315751 for *H. sweglesi*.

## References

[ece38019-bib-0001] Abe, K. T., Mariguela, T. C., Avelino, G. S., Foresti, F., & Oliveira, C. (2014). Systematic and historical biogeography of the bryconidae (ostariophysi: Characiformes) suggesting a new rearrangement of its genera and an old origin of mesoamerican ichthyofauna. BMC Evolutionary Biology, 14, 1–15. 10.1186/1471-2148-14-152 25005252 PMC4109779

[ece38019-bib-0002] Albornoz‐Garzon, J. G., Mendez‐Lopez, A., DoNascimiento, C., & Lima, F. C. T. (2019). A new species of *Hemigrammus* (Characiformes: Characidae) from western Amazon River basin, Colombia. Journal of Fish Biology, 95, 932–939. 31281997 10.1111/jfb.14091

[ece38019-bib-0003] Barreto, C. A. V., Granja, M. M. C., Vidigal, P. M. P., Carmo, A. O., & Dergam, J. A. (2017). Complete mitochondrial genome sequence of neotropical fish *Astyanax giton* Eigenmann 1908 (Ostariophysi; Characidae). Mitochondrial DNA Part B, 2, 839–840. 33474004 10.1080/23802359.2017.1403869 PMC7800921

[ece38019-bib-0004] Benson, D. A., Cavanaugh, M., Clark, K., Karsch‐Mizrachi, I., Ostell, J., Pruitt, K. D., & Sayers, E. W. (2018). GenBank. Nucleic Acids Research, 46, D41–D47. 10.1093/nar/gkx1094 29140468 PMC5753231

[ece38019-bib-0005] Bernt, M., Braband, A., Schierwater, B., & Stadler, P. F. (2013). Genetic aspects of mitochondrial genome evolution. Molecular Phylogenetics and Evolution, 69, 328–338. 10.1016/j.ympev.2012.10.020 23142697

[ece38019-bib-0006] Bernt, M., Donath, A., Jühling, F., Externbrink, F., Florentz, C., Fritzsch, G., Pütz, J., Middendorf, M., & Stadler, P. F. (2013). MITOS: Improved de novo metazoan mitochondrial genome annotation. Molecular Phylogenetics and Evolution, 69, 313–319. 10.1016/j.ympev.2012.08.023 22982435

[ece38019-bib-0007] Brandão‐Dias, P. F. P., Carmo, A. O., do Martins, A. P. V., Pimenta, R. J. G., Alves, C. B. M., & Kalapothakis, E. (2016). Complete mitochondrial genome of *Salminus brasiliensis* (Characiformes, Characidae). Mitochondrial DNA Part A, DNA Mapping, Sequencing, and Analysis, 27, 1577–1578. 10.3109/19401736.2014.958676 25208163

[ece38019-bib-0008] Brown, W. M. Jr, George, M., & Wilson, A. C. (1979). Rapid evolution of animal mitochondrial DNA. Proceedings of the National Academy of Sciences of the United States of America, 76, 1967–1971. 10.1073/pnas.76.4.1967 109836 PMC383514

[ece38019-bib-0009] Burland, T. G. (2000). DNASTAR's Lasergene sequence analysis software. Methods in Molecular Biology, 132, 71–91. 10547832 10.1385/1-59259-192-2:71

[ece38019-bib-0010] Buroker, N. E., Brown, J. R., Gilbert, T. A., O'Hara, P. J., Beckenbach, A. T., Thomas, W. K., & Smith, M. J. (1990). Length heteroplasmy of sturgeon mitochondrial DNA: An illegitimate elongation model. Genetics, 124, 157–163. 10.1093/genetics/124.1.157 1968410 PMC1203902

[ece38019-bib-0011] Calcagnotto, D., Schaefer, S. A., & Desalle, R. (2005). Relationships among characiform fishes inferred from analysis of nuclear and mitochondrial gene sequences. Molecular Phylogenetics and Evolution, 36, 135–153. 10.1016/j.ympev.2005.01.004 15904862

[ece38019-bib-0012] Camacho, L. R., Pozzi, A. G., de Freitas, E. G., Shimizu, A., & Pandolfi, M. (2020). Morphological and immunohistochemical comparison of the pituitary gland between a tropical *Paracheirodon axelrodi* and a subtropical *Aphyocharax anisitsi* characids (Characiformes: Characidae). Neotropical Ichthyology, 18, 190092. 10.1590/1982-0224-2019-0092

[ece38019-bib-0013] Chan, P. P., & Lowe, T. M. (2019). tRNAscan‐SE: Searching for tRNA Genes in Genomic Sequences. Methods in Molecular Biology, 1962, 1–14. 31020551 10.1007/978-1-4939-9173-0_1 PMC6768409

[ece38019-bib-0014] Chen, H. P., Li, S. H., Xie, Z. Z., Zhang, Y., Zhu, C. H., Deng, S. P., Li, G. L., & Huang, H. (2016). The complete mitochondrial genome of the *Piaractus brachypomus* (Characiformes: Characidae). Mitochondrial DNA Part A, DNA Mapping, Sequencing, and Analysis, 27, 1289–1290. 10.3109/19401736.2014.945560 25090392

[ece38019-bib-0015] Ciloglu, A., Ellis, V. A., Duc, M., Downing, P. A., Inci, A., & Bensch, S. (2020). Evolution of vector transmitted parasites by host switching revealed through sequencing of *Haemoproteus* parasite mitochondrial genomes. Molecular Phylogenetics and Evolution, 153, 106947. 10.1016/j.ympev.2020.106947 32866615

[ece38019-bib-0016] Cui, Z., Liu, Y., & Chu, K. H. (2010). Broader pattern of tandem repeats in the mitochondrial control region of Perciformes. Chinese Journal of Oceanology and Limnology, 28, 785–794. 10.1007/s00343-010-9091-5

[ece38019-bib-0017] da Silva, M. A., Perazzo, G. X., Kavalco, K. F., & Pasa, R. (2020). Shape diversity of the fish genus *Astyanax* Baird & Girard, 1854 (Teleostei, Characidae) in adjacent basins. Biologia, 76, 213–221.

[ece38019-bib-0018] DoNascimiento, C., Herrera‐Collazos, E. E., Herrera‐R, G. A., Ortega‐Lara, A., Villa‐Navarro, F. A., Oviedo, J. S. U., & Maldonado‐Ocampo, J. A. (2017). Checklist of the freshwater fishes of Colombia: A Darwin Core alternative to the updating problem. ZooKeys, 708, 25–138. 10.3897/zookeys.708.13897 PMC5674168 29118633

[ece38019-bib-0019] Faria, T. C., Bastos, D. A., Zuanon, J., & Lima, F. C. T. (2020). A new *Hyphessobrycon* (Characiformes: Characidae) of the Hyphessobrycon heterorhabdus species‐group from the Central Amazon basin, Brazil. Zootaxa, 4859, 275–284. 10.11646/zootaxa.4859.2.6 33056200

[ece38019-bib-0020] Faria, T. C., Lima, F. C. T., & Wosiacki, W. B. (2020). A New *Hyphessobrycon* (Characiformes: Characidae) from the Guiana Shield in Northern Brazil. Copeia, 108, 369–375. 10.1643/CI-19-311

[ece38019-bib-0021] Gong, L., Shi, W., Si, L. Z., Wang, Z. M., & Kong, X. Y. (2015). The complete mitochondrial genome of peacock sole *Pardachirus pavoninus* (Pleuronectiformes: Soleidae) and comparative analysis of the control region among 13 soles. Molecular Biology, 49, 408–417. 10.1134/S0026893315030061 26107900

[ece38019-bib-0022] Greiner, S., Lehwark, P., & Bock, R. (2019). OrganellarGenomeDRAW (OGDRAW) version 1.3.1: Expanded toolkit for the graphical visualization of organellar genomes. Nucleic Acids Research, 47, W59–W64. 10.1093/nar/gkz238 30949694 PMC6602502

[ece38019-bib-0023] Guimarães, E. C., Brito, P. S., Bragança, P. H. N., Santos, J. P., Katz, A. M., Costa, L. F. C., & Ottoni, F. P. (2020). Integrative taxonomy reveals two new cryptic species of Hyphessobrycon Durbin, 1908 (Teleostei: Characidae) from the Maracaçumé and middle Tocantins River basins, Eastern Amazon Region. European Journal of Taxonomy, 723, 77–107. 10.5852/ejt.2020.723.1145

[ece38019-bib-0024] Guimarães, E. C., Brito, P. S., Feitosa, L. M., Costa, L. F. C., & Ottoni, F. P. (2019). A new cryptic species of Hyphessobrycon Durbin, 1908 (Characiformes, Characidae) from the Eastern Amazon, revealed by integrative taxonomy. Zoosystematics and Evolution, 95(2), 345–360. 10.3897/zse.95.34069

[ece38019-bib-0025] Huelsenbeck, J. P., & Ronquist, F. (2001). MRBAYES: Bayesian inference of phylogenetic trees. Bioinformatics, 17, 754–755. 10.1093/bioinformatics/17.8.754 11524383

[ece38019-bib-0026] Isaza, J. P., Alzate, J. F., & Maldonado‐Ocampo, J. A. (2016). Complete mitochondrial genome sequence of *Grundulus bogotensis* (Humboldt, 1821). Mitochondrial DNA Part A, DNA Mapping, Sequencing, and Analysis, 27, 2076–2078. 10.3109/19401736.2014.982563 25405907

[ece38019-bib-0027] Kalyaanamoorthy, S., Minh, B. Q., Wong, T. K. F., von Haeseler, A., & Jermiin, L. S. (2017). ModelFinder: Fast model selection for accurate phylogenetic estimates. Nature Methods, 14, 587–589. 10.1038/nmeth.4285 28481363 PMC5453245

[ece38019-bib-0028] Katoh, K., & Standley, D. M. (2013). MAFFT multiple sequence alignment software version 7: Improvements in performance and usability. Molecular Biology and Evolution, 30, 772–780. 10.1093/molbev/mst010 23329690 PMC3603318

[ece38019-bib-0029] Kumar, S., Stecher, G., Li, M., Knyaz, C., & Tamura, K. (2018). MEGA X: Molecular Evolutionary Genetics Analysis across Computing Platforms. Molecular Biology and Evolution, 35, 1547–1549. 10.1093/molbev/msy096 29722887 PMC5967553

[ece38019-bib-0030] Kurabayashi, A., & Ueshima, R. (2000). Complete sequence of the mitochondrial DNA of the primitive opisthobranch gastropod *Pupa strigosa*: Systematic implication of the genome organization. Molecular Biology and Evolution, 17, 266–277. 10.1093/oxfordjournals.molbev.a026306 10677849

[ece38019-bib-0031] Leggatt, R. A., & Devlin, R. H. (2020). Fluorescent protein transgenesis has varied effects on behaviour and cold tolerance in a tropical fish (*Gymnocorymbus ternetzi*): Implications for risk assessment. Fish Physiology and Biochemistry, 46, 395–403. 10.1007/s10695-019-00725-3 31748988

[ece38019-bib-0032] Letunic, I., & Bork, P. (2021). Interactive Tree Of Life (iTOL) v5: An online tool for phylogenetic tree display and annotation. Nucleic Acids Research, 49, W293–W296. 10.1093/nar/gkab301 33885785 PMC8265157

[ece38019-bib-0033] Li, C. Y., Sun, Z. J., Feng, S. M., Jiang, J. F., Wu, H. M., Zhang, Z. G., Cai, C., & Wang, Y. C. (2016). The complete mitochondrial genome of *Hemigrammus bleberi* . Mitochondrial DNA Part A, DNA Mapping, Sequencing, and Analysis, 27, 4449–4450. 10.3109/19401736.2015.1089565 26544159

[ece38019-bib-0034] Liu, H. Y., Sun, C. H., Zhu, Y., Li, Y. D., Wei, Y. S., & Ruan, H. H. (2020). Mitochondrial genomes of four American characins and phylogenetic relationships within the family Characidae (Teleostei: Characiformes). Gene, 762, 145041. 10.1016/j.gene.2020.145041 32777523

[ece38019-bib-0035] Liu, H. Y., Xu, N., Zhang, Q. Z., Wang, G. B., Xu, H. M., & Ruan, H. H. (2020). Characterization of the complete mitochondrial genome of *Drawida gisti* (Metagynophora, Moniligastridae) and comparison with other Metagynophora species. Genomics, 112, 3056–3064. 10.1016/j.ygeno.2020.05.020 32454169

[ece38019-bib-0036] Liu, Y. F., Meng, F., Liu, B. J., Huang, Y. K., Wang, Q., & Zhang, T. (2019). The complete mitochondrial genome of *Paracheirodon axelrodi* (Characiformes: Characidae) and phylogenetic studies of Characiformes. Mitochondrial DNA Part B Resources, 4, 3824–3825. 33366205 10.1080/23802359.2019.1681307 PMC7707614

[ece38019-bib-0037] Mathubara, K., & Toledo‐Piza, M. (2020). Taxonomic study of Moenkhausia cotinho Eigenmann, 1908 and *Hemigrammus newboldi* (Fernandez‐Yepez, 1949) with the description of two new species of Moenkhausia (Teleostei: Characiformes: Characidae). Zootaxa, 4852, 1–40. 10.11646/zootaxa.4852.1.1 33056706

[ece38019-bib-0038] Mirande, J. M. (2009). Weighted parsimony phylogeny of the family Characidae (Teleostei: Characiformes). Cladistics, 25, 574–613. 10.1111/j.1096-0031.2009.00262.x 34879592

[ece38019-bib-0039] Mirande, J. M. (2019). Morphology, molecules and the phylogeny of Characidae (Teleostei, Characiformes). Cladistics, 35, 282–300. 10.1111/cla.12345 34622981

[ece38019-bib-0040] Nguyen, L. T., Schmidt, H. A., von Haeseler, A., & Minh, B. Q. (2015). IQ‐TREE: A fast and effective stochastic algorithm for estimating maximum‐likelihood phylogenies. Molecular Biology and Evolution, 32, 268–274. 10.1093/molbev/msu300 25371430 PMC4271533

[ece38019-bib-0041] Padhi, A. (2014). Geographic variation within a tandemly repeated mitochondrial DNA D‐loop region of a North American freshwater fish, Pylodictis Olivaris. Gene, 538, 63–68. 10.1016/j.gene.2014.01.020 24440244

[ece38019-bib-0042] Paz, F. P. C., Batista, J. D., & Porto, J. I. R. (2014). DNA Barcodes of Rosy Tetras and Allied Species (Characiformes: Characidae: *Hyphessobrycon*) from the Brazilian Amazon Basin. PLoS One, 9, e98603. 10.1371/journal.pone.0098603 24878569 PMC4039478

[ece38019-bib-0043] Perna, N. T., & Kocher, T. D. (1995). Patterns of nucleotide composition at fourfold degenerate sites of animal mitochondrial genomes. Journal of Molecular Evolution, 41, 353–358. 10.1007/BF01215182 7563121

[ece38019-bib-0044] Rozas, J., Ferrer‐Mata, A., Sánchez‐DelBarrio, J. C., Guirao‐Rico, S., Librado, P., Ramos‐Onsins, S. E., & Sánchez‐Gracia, A. (2017). DnaSP 6: DNA Sequence Polymorphism Analysis of Large Data Sets. Molecular Biology and Evolution, 34, 3299–3302. 10.1093/molbev/msx248 29029172

[ece38019-bib-0045] Sharma, A., Siva, C., Ali, S., Sahoo, P. K., Nath, R., Laskar, M. A., & Sarma, D. (2020). The complete mitochondrial genome of the medicinal fish, *Cyprinion semiplotum*: Insight into its structural features and phylogenetic implications. International Journal of Biological Macromolecules, 164, 939–948. 10.1016/j.ijbiomac.2020.07.142 32687902

[ece38019-bib-0046] Silva, D. M. Z. D., Utsunomia, R., Ruiz‐Ruano, F. J., Oliveira, C., & Foresti, F. (2016). The complete mitochondrial genome sequence of *Astyanax paranae* (Teleostei: Characiformes). Mitochondrial DNA Part B Resources, 1, 586–587. 33490410 10.1080/23802359.2016.1222251 PMC7800300

[ece38019-bib-0047] Sun, C. H., Liu, H. Y., Xu, N., Zhang, X. L., Zhang, Q., & Han, B. P. (2021). Mitochondrial genome structures and phylogenetic analyses of two tropical characidae fishes. Frontiers in Genetics, 12, 627402. 10.3389/fgene.2021.627402 33633787 PMC7901900

[ece38019-bib-0048] Veríssimo‐Silveira, R., Gusmão‐Pompiani, P., Vicentini, C. A., & Quagio‐Grassiotto, I. (2010). Spermiogenesis and spermatozoa ultrastructure in *Salminus* and *Brycon*, two primitive genera in Characidae (Teleostei: Ostariophysi: Characiformes). Acta Zoologica, 87, 305–313. 10.1111/j.1463-6395.2006.00243.x

[ece38019-bib-0049] Xu, R., Zhao, Z. X., Xu, P., & Sun, X. W. (2015). The complete mitochondrial genome of the silvertip tetra, *Hasemania nana* (Characiformes: Characidae). Mitochondrial DNA Part A, DNA Mapping, Sequencing, and Analysis, 26, 889–890. 10.3109/19401736.2013.861445 24409871

[ece38019-bib-0050] Yan, J. Y., Li, W. Y., Song, P. F., Li, Y., Feng, S., & Liu, D. X. (2020). Characterization of the complete mitochondrial genome of *Chrysomela vigintipunctata* (Coleoptera: Chrysomelidae). Mitochondrial DNA Part B Resources, 5, 1475–1476.

[ece38019-bib-0051] Yuan, M. L., Zhang, Q. L., Guo, Z. L., Wang, J., & Shen, Y. Y. (2015). The complete mitochondrial genome of *Corizus tetraspilus* (Hemiptera: Rhopalidae) and phylogenetic analysis of Pentatomomorpha. PLoS One, 10, e0129003. 10.1371/journal.pone.0129003 26042898 PMC4456165

[ece38019-bib-0052] Zhang, D., Gao, F., Jakovlić, I., Zou, H., Zhang, J., Li, W. X., & Wang, G. T. (2020). PhyloSuite: An integrated and scalable desktop platform for streamlined molecular sequence data management and evolutionary phylogenetics studies. Molecular Ecology Resources, 20, 348–355. 10.1111/1755-0998.13096 31599058

[ece38019-bib-0053] Zhang, Q. Z., Sun, C. H., Zhu, Y., Xu, N., & Liu, H. Y. (2020). Genetic diversity and structure of the round‐tailed paradise fish (*Macropodus ocellatus*): Implications for population management. Global Ecology and Conservation, 21, e00876. 10.1016/j.gecco.2019.e00876

[ece38019-bib-0054] Zhang, S., Cui, J., Xu, R., Xu, P., & Sun, J. (2016). The complete mitochondrial genome of *Paracheirodon axelrodi* (Characiformes: Characidae: Paracheirodon). Mitochondrial DNA Part A, DNA Mapping, Sequencing, and Analysis, 27, 230–231. 10.3109/19401736.2014.880903 24495136

