# Supplementary material for: Mitochondrial genomes of five Hyphessobrycon tetras and their phylogenetic implications
Source: Ecol Evol. 2021 Aug 11;11(18):12754–64. doi: 10.1002/ece3.8019 (PMC8462149; doi:10.1002/ece3.8019)
Supplement: Supplementary file 1 — Table S1 [file ECE3-11-12754-s001.docx]

**Table S1** The published mitogenomes of Characiformes, Cypriniformes, and Perciformes used in phylogenetic analyses

| Order | Family | Species | Accession no. | Length(bp) |
| --- | --- | --- | --- | --- |
| Characiformes | Acestrorhynchidae | *Acestrorhynchus sp.* | AP011981.1 | 16,758 |
|  | Alestiidae | *Phenacogrammus interruptus* | AB054129.1 | 16,652 |
|  | Anostomidae | *Leporinus elongatus* | KU980144.1 | 16,774 |
|  | Bryconidae | *Brycon nattereri* | MT428073.1 | 16,837 |
|  |  | *Salminus brasiliensis* | KM245047.1 | 17,721 |
|  | Characidae | *Aphyocharax rathbuni* | MT185594.1 | 16,678 |
|  |  | *Astyanax giton* | MF805815.1 | 16,643 |
|  |  | *Astyanax mexicanus* | AP011982.1 | 16,682 |
|  |  | *Astyanax paranae* | KX609386.1 | 16,707 |
|  |  | *Gephyrocharax atracaudatus* | MH636341.1 | 17,049 |
|  |  | *Grundulus bogotensis* | KM677190.1 | 17,123 |
|  |  | *Hasemania nana* | AB861475.1 | 16,581 |
|  |  | *Hemigrammus bleheri* | LC074360.1 | 17,021 |
|  |  | *Hyphessobrycon herbertaxelrodi* | MT769327.1 | 17,417 |
|  |  | *Hyphessobrycon megalopterus* | MT185596.1 | 16,773 |
|  |  | *Hyphessobrycon elachys* | MW315747 | 17,224 |
|  |  | *Hyphessobrycon flammeus* | MW315748 | 16,008 |
|  |  | *Hyphessobrycon pulchripinnis* | MW315750 | 17,020 |
|  |  | *Hyphessobrycon roseus* | MW315749 | 17,046 |
|  |  | *Hyphessobrycon sweglesi* | MW315751 | 16,080 |
|  |  | *Moenkhausia costae* | MW366831.1 | 15,811 |
|  |  | *Moenkhausia pittieri* | MW366832.1 | 17,204 |
|  |  | *Moenkhausia sanctaefilomenae* | MW407181.1 | 18,437 |
|  |  | *Nematobrycon palmeri* | MN861079.1 | 17,340 |
|  |  | *Oligosarcus argenteus* | MF805814.1 | 16,711 |
|  |  | *Paracheirodon axelrodi* | AB898197.1 | 17,100 |
|  |  | *Paracheirodon innesi* | KT783482.1 | 16,962 |
|  |  | *Prionobrama filigera* | MT185593.1 | 16,683 |
|  | Citharinidae | *Citharinus congicus* | AP011985.1 | 16,453 |
|  | Ctenoluciidae | *Ctenolucius hujeta* | AP011987.1 | 16,599 |
|  | Curimatidae | *Curimata mivartii* | KP025764.1 | 16,705 |
|  | Cynodontidae | *Hydrolycus scomberoides* | AP011989.1 | 16,548 |
|  | Erythrinidae | *Hoplias intermedius* | KU523584.1 | 16,629 |
|  | Gasteropelecidae | *Carnegiella strigata* | AP011983.1 | 17,852 |
|  | Hemiodontidae | *Hemiodopsis gracilis* | AP011990.1 | 16,731 |
|  | Hepsetidae | *Hepsetus odoe* | AP011991.1 | 16,803 |
|  | Lebiasinidae | *Lebiasina astrigata* | AP011995.1 | 16,899 |
|  | Serrasalmidae | *Piaractus brachypomus* | KJ993871.2 | 16,722 |
| Cypriniformes | Cyprinidae | *Cyprinus carpio* | KJ511883.1 | 16,584 |
| Perciformes | Lateolabracidae | *Lateolabrax japonicus* | KR780682.1 | 16,593 |
