# Supplementary material for: Mitochondrial genomes of five Hyphessobrycon tetras and their phylogenetic implications
Source: Ecol Evol. 2021 Aug 11;11(18):12754–64. doi: 10.1002/ece3.8019 (PMC8462149; doi:10.1002/ece3.8019)
Supplement: Supplementary file 2 — Table S2 [file ECE3-11-12754-s002.docx]

**Table S2** Mitogenomic organization of the five *Hyphessobrycon* fish

| Gene | Position | | Length(bp) | Orientation | Codon | | IGN (bp) |
| --- | --- | --- | --- | --- | --- | --- | --- |
|  | From | To |  |  | Start | Stop |  |
| tRNA-Phe | 1 | 68 | 68 | J |  |  | 0 |
|  | 1 | 68 | 68 | J |  |  | 0 |
|  | 1 | 71 | 71 | J |  |  | 0 |
|  | 1 | 68 | 68 | J |  |  | 0 |
|  | 1 | 71 | 71 | J |  |  | 0 |
| 12S | 69 | 1013 | 945 | J |  |  | 2 |
|  | 69 | 1018 | 950 | J |  |  | 2 |
|  | 72 | 1022 | 951 | J |  |  | 2 |
|  | 69 | 1018 | 950 | J |  |  | 2 |
|  | 81 | 1032 | 952 | J |  |  | 11 |
| tRNA-Val | 1013 | 1084 | 72 | J |  |  | 1 |
|  | 1018 | 1088 | 71 | J |  |  | 1 |
|  | 1022 | 1093 | 72 | J |  |  | 1 |
|  | 1018 | 1089 | 72 | J |  |  | 1 |
|  | 1032 | 1103 | 72 | J |  |  | 1 |
| 16S | 1085 | 2754 | 1670 | J |  |  | 2 |
|  | 1089 | 2761 | 1673 | J |  |  | 2 |
|  | 1094 | 2772 | 1679 | J |  |  | 2 |
|  | 1090 | 2762 | 1673 | J |  |  | 2 |
|  | 1104 | 2772 | 1669 | J |  |  | 2 |
| tRNA-Leu | 2753 | 2827 | 75 | J |  |  | 0 |
|  | 2761 | 2835 | 75 | J |  |  | 1 |
|  | 2771 | 2844 | 74 | J |  |  | 0 |
|  | 2762 | 2836 | 75 | J |  |  | 1 |
|  | 2773 | 2847 | 75 | J |  |  | 2 |
| ND1 | 2828 | 3796 | 969 | J | ATG | TAA | 2 |
|  | 2836 | 3804 | 969 | J | ATG | TAA | 2 |
|  | 2845 | 3816 | 972 | J | ATG | TAA | 2 |
|  | 2837 | 3808 | 972 | J | ATG | TAA | 2 |
|  | 2848 | 3819 | 972 | J | ATG | TAA | 2 |
| tRNA-Ile | 3807 | 3877 | 71 | J |  |  | 12 |
|  | 3809 | 3880 | 72 | J |  |  | 6 |
|  | 3827 | 3898 | 72 | J |  |  | 12 |
|  | 3819 | 3890 | 72 | J |  |  | 12 |
|  | 3824 | 3895 | 72 | J |  |  | 6 |
| tRNA-Gln | 3877 | 3947 | 71 | N |  |  | 1 |
|  | 3879 | 3948 | 70 | N |  |  | 0 |
|  | 3898 | 3968 | 71 | N |  |  | 1 |
|  | 3889 | 3959 | 71 | N |  |  | 0 |
|  | 3894 | 3964 | 71 | N |  |  | 0 |
| tRNA-Met | 4005 | 4075 | 71 | J |  |  | 59 |
|  | 3966 | 4035 | 70 | J |  |  | 19 |
|  | 3980 | 4049 | 70 | J |  |  | 13 |
|  | 3969 | 4038 | 70 | J |  |  | 11 |
|  | 3978 | 4048 | 71 | J |  |  | 15 |
| ND2 | 4078 | 5136 | 1059 | J | ATG | TAG | 4 |
|  | 4032 | 5084 | 1053 | J | ATG | TAA | -2 |
|  | 4051 | 5023 | 973 | J | ATG | T | 3 |
|  | 4040 | 5108 | 1069 | J | ATG | T | 3 |
|  | 4050 | 5131 | 1082 | J | ATG | T | 3 |
| tRNA-Trp | 5167 | 5239 | 73 | J |  |  | 32 |
|  | 5120 | 5190 | 71 | J |  |  | 37 |
|  | 5133 | 5203 | 71 | J |  |  | 111 |
|  | 5119 | 5188 | 70 | J |  |  | 12 |
|  | 5145 | 5214 | 70 | J |  |  | 15 |
| tRNA-Ala | 5243 | 5311 | 69 | N |  |  | 5 |
|  | 5228 | 5296 | 69 | N |  |  | 39 |
|  | 5212 | 5280 | 69 | N |  |  | 10 |
|  | 5196 | 5264 | 69 | N |  |  | 9 |
|  | 5221 | 5289 | 69 | N |  |  | 8 |
| tRNA-Asn | 5313 | 5385 | 73 | N |  |  | 3 |
|  | 5298 | 5369 | 72 | N |  |  | 3 |
|  | 5282 | 5354 | 73 | N |  |  | 3 |
|  | 5266 | 5338 | 73 | N |  |  | 3 |
|  | 5292 | 5361 | 70 | N |  |  | 4 |
| tRNA-Cys | 5416 | 5484 | 69 | N |  |  | 32 |
|  | 5401 | 5469 | 69 | N |  |  | 33 |
|  | 5385 | 5452 | 68 | N |  |  | 32 |
|  | 5368 | 5435 | 68 | N |  |  | 31 |
|  | 5400 | 5468 | 69 | N |  |  | 40 |
| tRNA-Tyr | 5485 | 5554 | 70 | N |  |  | 2 |
|  | 5470 | 5539 | 70 | N |  |  | 2 |
|  | 5453 | 5522 | 70 | N |  |  | 2 |
|  | 5436 | 5505 | 70 | N |  |  | 2 |
|  | 5469 | 5538 | 70 | N |  |  | 2 |
| COXⅠ | 5556 | 7115 | 1560 | J | ATG | AGG | 3 |
|  | 5541 | 7100 | 1560 | J | ATG | AGG | 3 |
|  | 5524 | 7083 | 1560 | J | ATG | AGG | 3 |
|  | 5507 | 7066 | 1560 | J | ATG | AGG | 3 |
|  | 5540 | 7099 | 1560 | J | ATG | AGG | 3 |
| tRNA-Ser | 7103 | 7174 | 72 | N |  |  | -11 |
|  | 7088 | 7159 | 72 | N |  |  | -11 |
|  | 7071 | 7142 | 72 | N |  |  | -11 |
|  | 7054 | 7125 | 72 | N |  |  | -11 |
|  | 7087 | 7157 | 71 | N |  |  | -11 |
| tRNA-Asp | 7179 | 7247 | 69 | J |  |  | 6 |
|  | 7163 | 7233 | 71 | J |  |  | 5 |
|  | 7148 | 7219 | 72 | J |  |  | 7 |
|  | 7129 | 7200 | 72 | J |  |  | 5 |
|  | 7161 | 7232 | 72 | J |  |  | 5 |
| COXⅡ | 7261 | 7951 | 691 | J | ATG | T | 15 |
|  | 7249 | 7939 | 691 | J | ATG | T | 17 |
|  | 7234 | 7924 | 691 | J | ATG | T | 16 |
|  | 7212 | 7902 | 691 | J | ATG | T | 13 |
|  | 7246 | 7933 | 688 | J | ATG | T | 15 |
| tRNA-Lys | 7952 | 8024 | 73 | J |  |  | 2 |
|  | 7940 | 8012 | 73 | J |  |  | 2 |
|  | 7922 | 7994 | 73 | J |  |  | -1 |
|  | 7900 | 7972 | 73 | J |  |  | -1 |
|  | 7934 | 8006 | 73 | J |  |  | 2 |
| ATP8 | 8028 | 8192 | 165 | J | ATG | AGG | 5 |
|  | 8016 | 8183 | 168 | J | ATG | TAG | 5 |
|  | 7996 | 8163 | 168 | J | ATG | TAG | 3 |
|  | 7974 | 8141 | 168 | J | ATG | TAG | 3 |
|  | 8008 | 8175 | 168 | J | ATG | TAG | 3 |
| ATP6 | 8176 | 8863 | 688 | J | ATG | T | -15 |
|  | 8168 | 8855 | 688 | J | ATG | T | -14 |
|  | 8148 | 8832 | 685 | J | ATG | T | -14 |
|  | 8132 | 8813 | 682 | J | ATG | T | -8 |
|  | 8160 | 8847 | 688 | J | CTG | T | -14 |
| COXⅢ | 8864 | 9649 | 786 | J | ATG | TAA | 2 |
|  | 8856 | 9641 | 786 | J | ATG | TAA | 2 |
|  | 8833 | 9618 | 786 | J | ATG | TAA | 2 |
|  | 8814 | 9599 | 786 | J | ATG | TAA | 2 |
|  | 8848 | 9633 | 786 | J | ATG | TAA | 2 |
| tRNA-Gly | 9648 | 9719 | 72 | J |  |  | 0 |
|  | 9640 | 9712 | 73 | J |  |  | 0 |
|  | 9617 | 9689 | 73 | J |  |  | 0 |
|  | 9598 | 9670 | 73 | J |  |  | 0 |
|  | 9632 | 9704 | 73 | J |  |  | 0 |
| ND3 | 9738 | 10070 | 333 | J | ATG | TAA | 20 |
|  | 9713 | 10063 | 351 | J | ATG | TAA | 2 |
|  | 9690 | 10040 | 351 | J | ATG | TAA | 2 |
|  | 9671 | 10021 | 351 | J | ATG | TAA | 2 |
|  | 9705 | 10055 | 351 | J | ATG | TAA | 2 |
| tRNA-Arg | 10069 | 10137 | 69 | J |  |  | 0 |
|  | 10062 | 10130 | 69 | J |  |  | 0 |
|  | 10039 | 10107 | 69 | J |  |  | 0 |
|  | 10020 | 10088 | 69 | J |  |  | 0 |
|  | 10059 | 10127 | 69 | J |  |  | 5 |
| ND4L | 10138 | 10432 | 295 | J | ATG | T | 2 |
|  | 10131 | 10427 | 297 | J | ATG | TAA | 2 |
|  | 10108 | 10404 | 297 | J | ATG | TAA | 2 |
|  | 10089 | 10385 | 297 | J | ATG | TAA | 2 |
|  | 10128 | 10424 | 297 | J | ATG | TAA | 2 |
| ND4 | 10427 | 11807 | 1381 | J | ATG | T | -4 |
|  | 10421 | 11801 | 1381 | J | ATG | T | -5 |
|  | 10398 | 11778 | 1381 | J | ATG | T | -5 |
|  | 10379 | 11759 | 1381 | J | ATG | T | -5 |
|  | 10418 | 11798 | 1381 | J | ATG | T | -5 |
| tRNA-His | 11808 | 11876 | 69 | J |  |  | 2 |
|  | 11802 | 11870 | 69 | J |  |  | 2 |
|  | 11779 | 11847 | 69 | J |  |  | 2 |
|  | 11760 | 11828 | 69 | J |  |  | 2 |
|  | 11799 | 11867 | 69 | J |  |  | 2 |
| tRNA-Ser | 11877 | 11944 | 68 | J |  |  | 2 |
|  | 11871 | 11938 | 68 | J |  |  | 2 |
|  | 11848 | 11915 | 68 | J |  |  | 2 |
|  | 11829 | 11896 | 68 | J |  |  | 2 |
|  | 11868 | 11935 | 68 | J |  |  | 2 |
| tRNA-Leu | 11946 | 12018 | 73 | J |  |  | 3 |
|  | 11940 | 12012 | 73 | J |  |  | 3 |
|  | 11917 | 11989 | 73 | J |  |  | 3 |
|  | 11898 | 11970 | 73 | J |  |  | 3 |
|  | 11937 | 12009 | 73 | J |  |  | 3 |
| ND5 | 12019 | 13857 | 1839 | J | ATG | TAA | 2 |
|  | 12013 | 13852 | 1840 | J | ATG | TAA | 2 |
|  | 11990 | 13828 | 1839 | J | ATG | TAA | 2 |
|  | 11971 | 13809 | 1839 | J | ATG | TAG | 2 |
|  | 12010 | 13845 | 1836 | J | ATG | TAA | 2 |
| ND6 | 13854 | 14369 | 516 | N | ATG | TAG | -2 |
|  | 13848 | 14360 | 513 | N | ATG | TAA | -3 |
|  | 13825 | 14340 | 516 | N | ATG | TAA | -2 |
|  | 13806 | 14318 | 513 | N | ATG | TAG | -2 |
|  | 13843 | 14356 | 514 | N | ATG | T | -1 |
| tRNA-Glu | 14370 | 14437 | 68 | N |  |  | 2 |
|  | 14361 | 14434 | 74 | N |  |  | 2 |
|  | 14342 | 14409 | 68 | N |  |  | 3 |
|  | 14319 | 14386 | 68 | N |  |  | 2 |
|  | 14357 | 14424 | 68 | N |  |  | 2 |
| Cytb | 14442 | 15578 | 1137 | J | ATG | TAA | 6 |
|  | 14434 | 15574 | 1141 | J | ATG | T | 1 |
|  | 14415 | 15551 | 1137 | J | ATG | TAA | 7 |
|  | 14390 | 15526 | 1137 | J | ATG | TAA | 5 |
|  | 14430 | 15566 | 1137 | J | ATG | TAA | 7 |
| tRNA-Thr | 15582 | 15652 | 71 | J |  |  | 5 |
|  | 15575 | 15646 | 72 | J |  |  | 2 |
|  | 15555 | 15627 | 73 | J |  |  | 5 |
|  | 15531 | 15602 | 72 | J |  |  | 6 |
|  | 15570 | 15642 | 73 | J |  |  | 5 |
| tRNA-Pro | 15651 | 15720 | 70 | N |  |  | 0 |
|  | 15645 | 15714 | 70 | N |  |  | 0 |
|  | 15626 | 15695 | 70 | N |  |  | 0 |
|  | 15601 | 15670 | 70 | N |  |  | 0 |
|  | 15641 | 15710 | 70 | N |  |  | 0 |
| CR | 15721 | 17224 | 1504 | J |  |  | 2 |
|  | 15715 | 16008 | 294 | J |  |  | 2 |
|  | 15696 | 17020 | 1325 | J |  |  | 2 |
|  | 15671 | 17046 | 1376 | J |  |  | 2 |
|  | 15711 | 16079 | 369 | J |  |  | 2 |

Note: The sequence of mitogenomes is as follows: *Hyphessobrycon elachys*, *Hyphessobrycon flammeus*, *Hyphessobrycon pulchripinnis*, *Hyphessobrycon roseus*, and *Hyphessobrycon sweglesi*. IGN (Intergenic nucleotides) was used to analyze genetic overlap.
